# Coping styles in HIV positives and HIV negatives

**DOI:** 10.1186/s40359-020-00484-4

**Published:** 2020-11-03

**Authors:** Mohammadreza Heydari, Yasaman Karimzadeh, Marjan Faghih, Zahra Heydari, Elhamsadat Hosseini, Morteza Mehraeen

**Affiliations:** 1 Shiraz HIV/AIDS Research Center (SHARC), Shiraz University of Medical Sciences, Shiraz, Fars Iran; 2 Department of Biostatistics, School of Medicine, Arak University of Medical Sciences, Arak, Iran; 3 Department of Biology, Shiraz University of Sciences, Shiraz, Iran; 4 Voluntary Counseling and Testing Center, 2nd floor, Lavan Ave, Delavaran-e Basij Blvd, Khatoun Sq, Shiraz, Fars Iran

**Keywords:** HIV, Coping styles, Etiology

## Abstract

**Background:**

Choosing the most useful and versatile way to solve one's personal and social problems is one of the most important choices in individual life. The aim of this study was to compare the coping styles of people living with Human immunodeficiency virus positive and negative.

**Methods:**

This is a Cross-sectional study that accomplished in Shiraz Behavioural Disease Counselling Centre in 2019 and 2020. For this purpose, in the first phase, 40 HIV+ and 40 HIV− patients were randomly selected to answer the questionnaire of dealing with the stressful conditions of Andler and Parker. In the second phase, the same questionnaire was filled out along with a reality distortion questionnaire from similar individuals (40 HIV+ and 40 HIV−).

**Results:**

92% of the HIV population in this study was between 15 and 55 years and 8% was upper than 55 years. 90% of them had no university degree. Among all, 47.5% of them were, 48.5% were self-employed and 49% of them were infected sexually. The results showed that in the first stage there was a significant relationship between marital status and the chances of getting the disease in people, and after controlling the demographic factors, coping styles did not show a significant effect on the disease. In the second stage, the factors of age, sex, education, and marital status had significant effects on people living with HIV, but the effect of coping styles on people with HIV was not significant (*P* < 0.05).

**Conclusion:**

Therefore, it can be concluded that demographic factors more than coping styles can affect the chances of high-risk behaviours; so, what is identified and measured as a coping style in people in the process that leads to the manifestation of high-risk behaviours or healthy behaviour does not matter much. It should be noted that the reason for rejecting the hypotheses of this study could be the effect of cultural and social factors of Iranian society.

## Background

"What we are today is the result of our past choices" [1] William Glaser, psychologist and the founder of Choice theory believed that the basic difference between humans is in their power of choice. The Choice theory leads us to the most fundamental human cognition. The choice of an appropriate coping style that could make formulation of the next choices is one of the most important decisions. How a person decides to cope with problems represents Self-Confidence, Self-esteem and expectation of himself and the environment [2, 3]. If he/she confronts the problem and tries to solve, it will be Problem Solving and if he/she gets away from it (like drinking, drug abuse, etc.), it will be emotional coping; also, if he/she escapes psychologically or physically, it will be Run [4]. Although our knowledge shows that Problem Solving has an association with resiliency and more successes in life [5, 6], there isn’t any evidence to prove which of them is better. For example, the best approach to endure grief, except through emotional coping. Rational Emotive Behaviour Therapy must begin while the mourning period lasts more than 2 weeks. Clearly, none of the defence mechanisms can be exactly useful or harmful; as Sigmund Freud said [7], extremist and long term use of these mechanisms can lead to maladaptive behaviours.

Numerous studies have confirmed the relationship between emotional well-being and behaviour such as alcohol, drug and Internet use, communication tensions and family problems, etc [8–10]. Other studies have shown the relationship between resilience and life skills with problem-solving [11]. Some research has even shown that the dominant coping style can be effective in positively and negatively influencing interpersonal communication [4]. This can clearly lead to the etiology of high-risk behaviours in individuals. The emotional and then Run people are more likely to engage in risky behaviours. Since high-risk behaviours are directly linked to Human immunodeficiency virus (HIV) in people [12], specific coping styles can predict HIV.

This study aimed to compare the coping styles in HIV positive versus HIV negative individuals.

## Methods

### Participants and research plan

In this cross-sectional study, the sample size was estimated using previous studies [13] and the following formula. Forty HIV positive subjects that had referred to Shiraz Behavioural Disease Counselling Centre and 80 HIV negative participants that had no history of high-risk behaviours were randomly selected.

$$\mathrm{n}=\frac{\left({\mathrm{S}}_{1}^{2}+{\mathrm{S}}_{2}^{2}\right)({{\mathrm{z}}_{1-\frac{\mathrm{\alpha }}{2}}+{\mathrm{z}}_{1-\upbeta })}^{2}}{{\mathrm{d}}^{2}}$$

$$d={\mu }_{1}-{\mu }_{2}=1.7 ,\alpha =.05, 1-\beta =0.8$$

$${z}_{1-\frac{\alpha }{2}}=1.96,{z}_{1-\beta }=0.84,{S}_{1}=5.439,{S}_{2}=5.398$$

### Procedure

Initially, the Andler and Parker Counter Styles Questionnaire was completed by 80 people (40 people HIV+ and 40 people HIV−) and the information was reviewed. However, the collected data was in conflict with the researcher’s expectation, so that we decided to repeat the test in order to examine the honesty of the responders. This part of the study done in 3 weeks.

In the second step, 10 questions from the ''reality-distortion questionnaire'' were added to the coping style questionnaire. The questionnaire was once again given to 40 people with HIV+ and 40 people without HIV in order to examine the relationship between coping styles and people with HIV. This part is done in 2 weeks. Totally 5 months.

#### Inclusion criteria

For this study, we needed 20 males and 20 females that infected HIV regardless of the way of infection (sexually, injection, tattoo, etc.) who were treated more than 1 year by anti-retroviruses drugs without age limit. Researchers didn’t check the mental status of patients.

#### Exclusion criteria

The HIV patients that didn’t complete the full questionnaire or answered by chance or questionnaire was distorted or did not want to participate were excluded.

#### Tools

(1) The Steller and Parker Stress Coping Styles Questionnaire has 48 questions scored using a five-point Likert scale, with three dimensions: (1) task ordinary coping style or actively dealing with the problem to manage it; (2) emotion-oriented coping style or focusing on emotional responses to the problem; (3) measure of Run coping style or avoidance of the problem. Questions 1, 2, 6, 10, 15, 21, 24, 26, 27, 36, 39, 41, 42, 43, 46, 47 are for problem solving and 5, 7, 8, 13, 14, 16, 17, 19, 22, 25, 28, 30, 33, 34, 38, 45 are for emotional ordinary and 3, 4, 9, 11, 12, 18, 20, 23, 29, 31, 32, 35, 37, 40, 44, 48 are for Run style.

Cronbach's alpha of the reliability of this questionnaire in all three factors is between 0.82 and 0.92. The validity coefficient of the questionnaire with stressful situations is obtained through Cronbach's alpha in Qurayshi research at a high level of 0.8133. The validity correlation coefficient of the questionnaire scales is equal to the task-oriented (0.58) and the emotion-oriented (0.55) and the avoidance-oriented (0.33). One point was considered for each questionnaire item and individual coping style was defined as the highest score between the three dimensions [14].

(2) The reality distortion questionnaire was developed in 2001 by Carpenters and Sudanese at Shahid Chamran University in Ahvaz, Iran. This questionnaire has 10 questions, with answers based on the 5-point Likert scale. The answer score varies between 10 and 50. The internal correlation coefficient of this questionnaire was 0.75 and its retesting coefficients were 0.55, 0.40, 0.31, and 0.41, compared to Eysenck lie detector, MMPI, Cooper Smith, and Crowne and Marlowe [15].

### Statistical analysis

Data collected from both groups were analysed using SPSS software version 23. The Kolmogorov Smirnov test was used to examine the normality of the data, and a mean comparison of a small factor between the two groups was performed with an independent t-test. The relationship between the classification factors was investigated using the chi-square test or Fisher exact test. Multiple and binary logistic regression were used to investigate the single and simultaneous effect of the coping style factors on HIV infected people.

## Results

We investigated the relationships between the factors without considering distortion of reality.

As the Table 1 shows, the mean age of HIV+ individuals was significantly higher than those tested HIV− (*P* < 0.05). Additionally, most people tested HIV+ had undergraduate education (90%), whereas only 75% of those individuals tested HIV− had education above the diploma level. A significant relationship between marital status and disease was also identified (*P* < 0.05).

**Table 1:** Demographic characteristics of the two groups (HIV− and HIV+)

Related factors	HIV+ (n = 40)	HIV− (n = 40)	Total	Statistical index	Sig *P *value
Age mean ± SD	42.12 ± 5.84	35.10 ± 75.4	39.12 ± 13.1	t = 2.59	0.01
Gender					
Male	20 (50)	20 (50)	40 (50)	ͯ^2^ = 0.000	1
Female	20 (50)	20 (50)	40 (50)		
Education					
Without academic education	36 (90)	10 (25)	46 (57.5)	ͯ^2^ = 58.34	*P* ˂ 0.0001
Academic education	4 (10)	30 (75)	34 (42.5)		
Marital status					
Single	8 (20)	19 (47.5)	27 (33.8)	z^2 ^= 79.14	0.001
Married	19 (47.5)	20 (50)	39 (48.8)		
Divorced/widowed	13 (32.5)	1 (2.5)	14 (17.5)		

T = Independent *t* test; χ^2^ chi-square test

As the Table 2 shows, coping styles may not be a good predictor of disease (*P* < 0.051), but age, education, and marital status had a significant effect on the disease (*P* < 0.05). Thus, the effect of coping styles on the disease was analysed using the multiple models by controlling the effect of age, education and marital status; the results still did not show a significant effect of coping styles after controlling demographic factors (*P* = 0.053).

**Table 2:** Results of logistic regression analysis on the effect of coping styles on HIV infection after controlling the effect of demographic factors

Factor	HIV+ (N = 40)	HIV− (N = 40)	Univariate		Multiple	
			OR (95% CI)	*P *value	OR (95% CI)	*P *value
Coping style						
Task-oriented	28 (70)	29 (72.5)	0.89 (0.2–4.33)	0.81	7–0.09 1.61 (0.36)	0.53
Emotion-oriented	12 (30)	11 (27.5)	1		1	
Age						
–	42.12 ± 5.8	3.10 ± 75.4	1–0.09 1.05 (1.01)	0.02	1–0.01 0.03 (9.97)	0.29
Gender						
Male	20 (50)	20 (50)	1 (0.2–42.4)	1	-	-
Female	20 (50)	20 (50)	1		-	
Education						
Without academic education	36 (90)	10 (25)	27 (7.94–7.9)	*P* < 0.001	99–0.6 23.98 (5.77)	*P* < 0.001
Academic education	4 (10)	35 (75)	1		1	
Marital status						
Single	8.20	19 (47.5)	1	–	1	-
Married	19 (47.5)	(20/50)	2.25 (0.6–8.37)	0.124	13–0.9 3.48 (0.87)	0.08
Divorced/widowed	13 (32.5)	1 (2.5)	277–0.3 30.88 (3.45)	0.002	224–19.6 6 (1.58)	0.02

Ref = reference group; OR (95% CI) = 95% confidence interval for OR

We investigated the relationships between the factors by considering the distortion of reality.

By entering reality distortion questions, 18 out of 80 participants (22.5%) were identified as distorting individuals. Of these 18, only 2 (2.5%) were HIV− positive and 16 (20%) were HIV+. Therefore, at this stage, the analysis of relationships was performed on the remaining 62 participants.

As the Table 3 shows, the age of people living with HIV− is significantly higher than those with HIV+. More significantly, those with HIV+ are men, and those living with HIV− are women (*P* < 0.05). Equally revealing is that more educated people were in the two groups (HIV− and HIV+). Also, those married, divorced or widowed were more likely to be within these two groups as opposed to single people (*P* < 0.05).

**Table 3:** Comparison of demographic characteristics of the age groups (HIV− and HIV+)

Factor	HIV+	HIV−	Total		*P *value
Age	36.7 ± 04.26	41.7 ± 42.56	39.7 ± 27.87	T = 2.74	0.008
Gender					
Male	14 (58.3)	7 (19.41)	21 (35)	ͯ^2^ = 9.57	0.002
Female	10 (41.7)	29 (80.6)	39 (65)		
Education					
Without academic education	8 (34.8)	1 (2.6)	9 (14.8)	ͯ^2^ = 11.78	0.001
Academic education	15 (65.2)	37 (97.4)	52 (85.2)		
Marital status					
Single	8 (33.3)	10 (27.8)	18 (30)	Z = 5.87	0.039
Married	11 (45.8)	25 (69.4)	36 (60)		
Divorced/widowed	5 (20.8)	1 (2.8)	6 (10)		

T = Independent t-test; χ^2^ chi-square test; z = Fisher’s exact test

According to the Table 4, the univariate logistic regression model used to investigate the effect of individual coping styles showed that coping styles did not have a significant effect on those diagnosed with HIV+ (*P* < 0.053) regarding age, sex, education, and marital status factors. However, significant effects on people living with HIV were identified (*P* < 0.05). Therefore, in multiple logistic regression, after taking into account the effect of age, sex, education, and marital status factors, the impact of coping styles on HIV infected was investigated and only the difference in coping styles was not significant (*P* < 0.05).

**Table 4:** Results of logistic regression analysis on the effect of coping styles on HIV infection after controlling the effect of demographic factors

Related factors	HIV+ (N = 40)	HIV− (N = 40)	Univariate		Multiple	
			OR (95% CI)	*P *value	OR (95% CI)	*P *value
Coping style						
Emotion-oriented	19 (79/2)	33 (86/8)	0.58 (0.2–15.25)	0.43	25–0.3 2.94 (0.34)	0.33
Task-oriented	5 (20.8)	5 (13.2)	1		1	
Age						
–	36.7 ± 04.26	41.7 ± 42.56	0.9 (0.0–84.98)	0.012	0–0.94 0.38 (074)	0.003
Gender						
Male	14 (58.3)	7 (19.4)	5.8 (1.18–8.5)	0.003	21–0.6 4.29 (0.85)	0.08
Female	10 (41.7)	29 (80.6)	1		1	
Education						
Without academic education	8 (34.8)	1 (2.6)	19.73 (3.2–0.171)	0.007	15–0.22 61.53 (2.5)	0.02
Academic education	15 (65.2)	37 (97.4)	1		1	
Marital status						
Single	8 (33.3)	10 (27.8)	0.16 (0.1–02.7)	0.13	0.131–0.84 0.085 (0.004)	0.12
Married	11 (45.8)	25 (69.4)	0.09 (0.0–01.84)	0.04	2–0.56 0.14 (0.008)	0.19
Divorced	5 (20.8)	1 (2.8)	1	0.09	1	0.29

Ref = reference group; OR (95% CI) = 95% confidence interval for OR

## Discussion

In this study, it was assumed that what led people to a successful or unsuccessful life was how they analysed different situations and their choices, as Glaser believed [1]. Based on this assumption, we selected two groups of people with different lifestyles who should indicate the difference between the two lifestyles. Empirical evidence strongly suggests that HIV is most often the result of high-risk behaviours such as joint injections, unprotected sex, and so on. Conversely, a number of patients have been victims of physical and sexual abuse or rape, trust in their spouses, and ignorance of how HIV could be contracted. Therefore, HIV infection may not necessarily be the result of a specific type of lifestyle, which may explain why the findings of this study are inconsistent with those of many others [9, 16]. Moreover, it can be said that no human is 100% emotional or 100% problem-solving in life. Therefore, when Andler's questionnaire asks them about life situations, they both remember their different coping style.

In contrast, a brief look at the population of specialists and educated people involved in drug and alcohol addiction revealed that these people may have been problematic during their studies, but not so at other times [17]. Or when we examined the life of an HIV positive sex worker, we found notwithstanding the side effects of antiretroviral drugs, he/she is using them in his/her life. Thus, maybe he/she wasn't always emotional or Run.

In various articles, there are contradictory and often promising statistics on the extent to which people are aware of HIV and how it is contracted [18–20]. However, the results of this study highlighted that all the participants, despite engaging in what is considered low-risk behaviour, were unaware of how they were infected by their spouses or sexual partners.

According to social workers from the Counselling Canter for Behavioural Diseases, a large number of these people stated they would not have contracted these diseases if they had not lived in an inappropriate family and in the wrong environment. Therefore, in the context of using scientific and specialized findings, we should not always look for a meaningful difference in order to label a group or try to place a specific group within a superior position.

In both stages of the study marital status was identified as a main risk factor in HIV infection. Furthermore, injection is recognized as the main transfer method of HIV infection in Iran. Thus it can be concluded that married women have fallen victim through their drug injecting spouses, accordingly, no significant association was observed with women’s coping style in the current study.

Therefore, women get infected because they believe in their husbands’ health, more than their lifestyle. So; these findings did not confirm the findings of the articles with significant differences between both genders regarding their coping style choice [21–23]. On the other hand in comparison to more than the past 100 years, women participate more in social interaction in society, they are more accustomed to solving their problems at the time. Therefore, perhaps it would be better to use newer and more accurate tools that do not highlight gender or class differences to question the gap between the two sexes that has been inherited by default and to conduct newer research.

### Limitations

Low sample size was amongst limitations in this study, however, re-implementation of the study using lie detector has been supposed to cover the problem. Also because of the heterogeneity in the case group (injection addictions, sex workers and homeless), founding the homogeneous group as a control group was difficult.

About 5% of the patients were illiterate and their questions were filled by a trained psychologist in a face-to-face interview while other questionnaires were completed by the patients, themselves.

This questionnaire was not able to find random answers or answers that give a better social image to participants. So it's suggested to use clinical interviews and retrospective reports of the patient's life besides filling questionnaires.

In this study, we needed to all patients with all ways of getting infected. Therefore, we did not separate homeless and addicted patients and sex workers. In addition, we did not filter them in terms of mental and physical health. For future studies, a homogenous sample size of addicted or sex worker HIV positives patients is suggested.

## Conclusion

The rejection of this study’s hypotheses confirms that today’s psychological research requires a more in-depth and gender-neutral approach. These findings tell us that science may poison our beliefs just with a mistake.

## Data Availability

The datasets during and/or analysed during the current study available from the corresponding author on reasonable request.

## Abbreviation

HIV: Human immunodeficiency virus
